# Comprehensive Exploration of the Growth and Lipid Synthesis Phases of *T. oleaginosus* Cultures Implementing Design of Experiments and Response Surface Methodology

**DOI:** 10.3390/bioengineering10121359

**Published:** 2023-11-27

**Authors:** Vasileios Parisis, Olga Tsave, Christina Papanikolaou, Erasmia Pantazopoulou, Christos Chatzidoukas

**Affiliations:** Department of Chemical Engineering, Aristotle University of Thessaloniki (AUTH), 54124 Thessaloniki, Greece; parisisv@cheng.auth.gr (V.P.); tsaveolga@auth.gr (O.T.); christpg@cheng.auth.gr (C.P.); epantazo@hotmail.com (E.P.)

**Keywords:** *Cryptococcus curvatus*, oleaginous yeast, microbial lipids, lipogenesis, design of experiments, response surface methodology (RSM), media composition optimization, inorganic nitrogen source, C/N ratio parameterization

## Abstract

*Trichosporon oleaginosus* is an unconventional oleaginous yeast distinguished by its remarkable capacity to accumulate lipids in excess of 70% of its dry weight, particularly when cultivated in nitrogen-restricted conditions with ample carbon sources. A pivotal question that arises pertains to the nutrient dynamics in the culture medium, which give rise to both the excessive lipid content and corresponding lipid concentration. While previous research has predominantly focused on evaluating the impact of the initial carbon-to-nitrogen (C/N) ratio on lipid production, the precise critical thresholds of glucose and ammonium sulfate ((NH_4_)_2_SO_4_) at which growth and intracellular lipid production are either stimulated or impeded remain inadequately defined. This study employs an experimental design and response surface methodology to investigate the complex mechanism of lipid accumulation and its interaction with cellular growth. Application of the aforementioned methodologies resulted in the production of 10.6 g/L of microbial oil in batch cultures under conditions that correspond to a C/N ratio of 76. However, the primary objective is to generate knowledge to facilitate the development of efficient fed-batch cultivation strategies that optimize lipid production exclusively employing inorganic nitrogen sources by finely adjusting carbon and nitrogen levels. The intricate interaction between these levels is comprehensively addressed in the present study, while it is additionally revealed that as glucose levels rise within a non-inhibitory range, lipid-free biomass production decreases while lipid accumulation simultaneously increases. These findings set the stage for further exploration and the potential development of two-stage cultivation approaches, aiming to fully decouple growth and lipid production. This advancement holds the promise of bringing microbial oil production closer to commercial viability.

## 1. Introduction

Microbial lipids, also referred to as single-cell oils (SCOs), synthesized from oleaginous microorganisms, represent an increasingly promising resource for the biofuel and chemical industry. Utilizing microbial oils, particularly for biofuel production, offers several distinct advantages, including their resemblance to vegetable oils, which facilitates their downstream processing using existing technologies, and higher production rates. Moreover, given the potential of the oleaginous microorganisms to grow on carbon sources derived through the valorization of diverse waste streams, intensifies the ethical and environmental appeal of the corresponding microbial lipid production system and offers the advantage of independence from climate and/or seasonal changes as opposed to the reliance on energy crops typically associated with first- and second-generation biofuel production [1,2,3,4,5,6,7]. However, the industrial exploitation of oleaginous yeasts has yet to be fully explored due to considerable expenses, which are inversely associated with the productivity of the fermentation process [8]. Despite the potential to use hydrolysates derived from cost-effective carbon feedstocks, such as biomass or catering service wastes as substrates for microbial cultures, the production costs still pose a significant barrier [8].

Oleaginous yeasts are known to have the capacity to accumulate up to 70% *w*/*w* oil on a dry basis [9]. However, this characteristic alone does not guarantee economically sustainable microbial oil production unless accompanied by substantial culture productivity, yield, and consistent performance. Therefore, a profound understanding of oleaginous yeast physiology metabolism and the factors influencing lipogenesis under varying culture conditions is deemed essential for optimizing process performance and achieving economic viability.

Lipogenesis in oleaginous yeasts is a non-growth-associated metabolic process leading to intracellular oil accumulation. This process is influenced by various environmental variables and cellular states. Key factors significantly impacting the overall functioning and performance of oleaginous yeast cultures include the type and quantity of the carbon and nitrogen sources in the culture medium, the carbon-to-nitrogen (C/N) ratio, oxygen levels, temperature, pH, time of incubation, and mineral salt concentration among others [10]. Notably, nitrogen limitation has been identified as a critical parameter triggering a switching of the carbon metabolism and regulating the initiation of lipid accumulation in oleaginous microorganisms [11,12]. There have been several published studies in the open literature exploring the role of the carbon-to-nitrogen ratio and its implication along the culture growth of various oleaginous yeast strains [13,14,15,16,17,18,19]. Nonetheless, the research in this field remains incomplete and potentially misleading as there exist additional metabolically significant factors, including the absolute concentrations of each nutrient and conditions that must be concurrently considered to accurately characterize the factors governing the operation of the microbial oil production process. Therefore, the optimization of this process through the systematic parameterization of key process variables stands as a fundamental step toward ensuring the sustainability of oleaginous yeast cultures as single-cell oil (SCO) production units. This optimization aims to holistically enhance production rates, lipid yields, and contents.

In the present work, *Trichosporon oleaginosus* was selected as the model organism, with the goal of establishing a systematic framework to support the optimal operation and successful transition from batch to fed batch and continuous operating modes in SCO production units. The selection of this particular yeast was guided by a multitude of advantageous characteristics that pertain to the yeast’s physiology, including rapid growth, high lipid accumulation capacity, compatibility with a broad variety of substrates, ease of cultivation under cost-effective conditions, and notable resistance to inhibitors produced during lignocellulosic biomass pretreatment [10,20,21]. Moreover, *T. oleaginosus* presents high genetic modification capabilities rendering it a promising platform for custom tailored microbial oil production [22,23].

In most published studies on oleaginous yeast cultures, the use of organic nitrogen sources, such as yeast extract, either alone or complementary to an inorganic one (e.g., (NH_4_)_2_SO_4_), is prevalent [15,24,25]. However, the significantly high price of the yeast extract encourages the development of fermentation media based on cost-effective inorganic nitrogen sources. Τhe present study investigates *T. oleaginosus* cultures as an SCO production system utilizing solely inorganic nitrogen sources and aspires to address some critical questions to facilitate the design of optimal fed batch and/or continuous feeding strategies. The decision to use an inorganic nitrogen source was driven by both the cost-effectiveness and desire for a thorough examination of nitrogen’s influence on cell growth. This choice aimed to eliminate potential confounding effects associated with other growth factors present in more complex nitrogen sources, as is frequently observed with yeast extracts. Notably, while previous works in the literature have primarily focused on the initial C/N ratio’s effect on lipogenesis [15,24,25], the exact critical levels of glucose and (NH_4_)_2_SO_4_, at which growth and/or the intracellular product formation are triggered or inhibited, remain insufficiently delineated. The motive of this work is based on the fact that besides the nitrogen source type (inorganic vs. organic), the initial concentrations that formulate the C/N ratio in time during the whole period of the cultivation process (dynamically change in time) further influence lipid production, especially when taking into account inhibition phenomena, such as in the case of the glucose concentration (glucose inhibition). Hence, response surface methodology is adopted to explore the maximum effective glucose and (ΝH_4_)_2_SO_4_ concentrations during fermentation processes with the selected yeast and to feature how their interplay impacts the extent of the lipid’s biosynthesis mechanism. To undertake this comprehensive investigation, a full factorial central composite design of experiments was implemented to assess the impact of different media compositions in cultures conducted at the flask scale. The experimental investigation was conducted under conditions of nutrient sufficiency for other important constituents, such as phosphorus (P), potassium (K), and sulfur (S), among others, to exclude or minimize the likelihood of these additional nutrients affecting the culture performance in variable ways. Furthermore, commercial glucose was deliberately chosen as the sole carbon source, instead of a complex mixture that might have originated from waste processing, to eliminate unaccounted factors that could potentially introduce unexplained variability in the experimental data and impede the comprehension over fundamental culture conditions that influence the physiology and the mechanisms underlying oil synthesis in the chosen yeast strain. The statistical analysis of the experimental data facilitated the establishment of operational boundaries within the process, particularly delineating the ranges of glucose and (NH_4_)_2_SO_4_ concentrations. This analysis unveiled unanticipated trade-offs between the growth and lipid synthesis aspects within *T. oleaginosus* cultures. Subsequently, the acquired insights were expanded upon and validated by replicating specific experimental conditions in cultures conducted within meticulously controlled bioreactor units.

## 2. Materials and Methods

### 2.1. Strain, Inoculum, and Media

The oleaginous yeast *Trichosporon oleaginosus* ATCC 20509 (formerly known as *Cryptococcus curvatus*), was employed in this study. The microorganism was cryopreserved at −80 °C in microtubes containing 1:1 YPD medium and 45% *v/v* glycerol. The composition of the YPD medium was (in g/L) as follows: yeast extract 10; peptone 10; D-glucose 20. The inoculum was prepared following a sequence of two successive pre-cultures in an orbital shaker. During the first preculture stage, the thawed cells were streaked on nutrient broth agar plates and incubated in an orbital shaker (BioBase, BJPX-2012R) at 30 °C until the formation of single colonies (within approximately 24–48 h). In the second step, a single colony was transferred from the agar plate in a 500 mL baffled-bottom conical flask with 250 mL of YPD medium, using an inoculating loop, and incubated at 180 rpm and 30 °C overnight until the optical density (OD_@600nm_) lied in the 1.5–2.5 range. An appropriate volume of the second preculture was used as the inoculum for the main cultures, after centrifugation (Megafuge 1.0 R, Heraeus) and resuspension in fresh culture medium to achieve an OD_@600nm_ of 1.5–2.5. A typical main culture medium composition was (in g/L) as follows: KH_2_PO_4_ 7; Na_2_HPO_4_ 2.5; FeCl_3_·6H_2_O 0.03; MgSO_4_·7H_2_O 0.3; MnCl_2_ 0.014; ZnSO_4_·H_2_O 0.06; CaCl_2_·2H_2_O 0.003. Glucose and (ΝH_4_)_2_SO_4_ concentrations are given for each experimental trial in the following sections.

### 2.2. Design of Experiments

In this study, we employed response surface methodology (RSM) with the implementation of a face-centered central composite design of experiments. The primary objective was to systematically explore the relationship between key performance indicators (KPIs) of the process and selected process variables, with a focus on uncovering potential interactions among these variables. The overarching goal was to optimize these KPIs by identifying the optimal combination of process variables. The two primary process variables under examination (factors) were the concentrations of glucose and inorganic nitrogen, sourced from (ΝH_4_)_2_SO_4_ within the culture medium. The following process responses, which served as performance criteria (KPIs) were examined through the implemented RSM: total biomass concentration (g/L), lipid content (g/g), and lipid free (residual) biomass concentration. The design adopted for this experimentation encompassed a 2^2^ factorial design augmented using a set of center points of which coordinates are given by the medians of the values of the factors used in the factorial part of the design. Additionally, a set of axial points strategically located at the center of each face of the design space completed the experimental configuration. Overall, this design yielded a total of 13 distinct experimental trials distinguished by varying levels of glucose and inorganic nitrogen (comprising three levels per factor). Detailed experimental conditions are outlined in Table 1.

The experiments were performed in 250 mL baffled-bottom flasks with a working volume of 120 mL. The flasks were kept in an incubator at a constant temperature of 30 °C and agitation of 150 rpm. A silicon tube ending to a nozzle was immersed through the hydrophobic cotton cap of the flasks into the medium. An air feeding stream was introduced through this tube to ensure culture aeration at a constant rate of 0.83 vvm. All the 13 experiments commenced simultaneously, utilizing the same preculture for inoculation, and were sustained over a duration of 120 h. For each performance criterion, a response surface curve was generated using second-order polynomials given by Equation (1):

(1) $$Y=\beta_{0}+\sum \beta_{i}X_{i}+\sum \beta_{ii}X_{i}^{2}+\sum \beta_{ij}X_{i}X_{j}$$

where *Y* is the response variable, *β*_0_ is the offset term, *β_i_* is the linear effect coefficient, *β_ii_* represents the quadratic effect coefficient, and *β_ij_* denotes the *ij*th order interaction effect coefficient; *X_i_* and *X_j_* represent the input variables (factors) that affect the response *Y*. Analysis of variance (ANOVA) was performed to evaluate the adequacy of the produced models, whereas the significance of the second-order equations and the individual terms was assessed using Fisher’s F-test. All calculation activities were performed using the Minitab 21^®^ statistical software package.

### 2.3. Bioreactor Culture Conditions

In addition to the planned flask-scale factorial experiments, this study encompassed experimental procedures conducted in a 3 L bioreactor (BIONET, F0-BABY) with a working volume of 2 L. The bioreactor system is equipped with two Rushton blade impellers, an aeriation system employing a ring sparger, and instrumentation comprising a pH probe (Hamilton, EasyFerm Bio PHI Arc 325), a dissolved oxygen (D.O.) probe (Hamilton, VisiFerm DO Arc 325 H0), an optical density (O.D.) probe (Optek, ASD12-N Absorption Probe), and an exhaust gas monitoring system (BIONET, bBReath4). A temperature control was implemented to maintain a constant temperature of 30 °C within the bioreactor. Heating and cooling were provided to the bioreactor by virtue of an electric heating jacket and an internal cooling finger, respectively. The pH was maintained at 6 ± 0.2 via the automatic addition of a 2 M NaOH solution. To promptly address any foam formation, a level transmitter was employed, and the issue was effectively mitigated by the addition of a 2% antifoam dispersion (Antifoam 204, Sigma-Aldrich, Saint Louis, MO, USA). The dissolved oxygen level was set at 20% of its saturation value and was controlled by manipulating the agitation rate and culture aeriation according to a cascade PI control law. The initial aeriation rate was set to 0.3 L/min, ensuring the necessary supply of oxygen for the biological processes.

### 2.4. Analytical Methods

The monitoring of key process variables involved the collection of 10–15 mL aliquots from the culture broth, regularly for the bioreactor scale experiments and at the end of the cultivation period for the flask-scale experiments. The optical density was determined by measuring the culture absorbance at 600 nm with a Vis spectrophotometer (Hach DR 3900). Subsequently, the collected samples were centrifuged at 4015× *g* (rcf) for 10 min, followed by a washing step with distilled H_2_O, and then a re-centrifugation step. The resulting biomass pellets were lyophilized for 18 h (ScanVac, CoolSafe 55-9 De-Ice), and the supernatant was collected for glucose and ΝH_4_^+^ determinations. The quantification of the dry cell weight was accomplished through precise weighing of the lyophilized biomass with an analytical balance. For determination of the glucose concentration in the supernatant, a commercial biochemistry analyzer (Xylem, YSI 2900) equipped with a glucose membrane was employed. The sample volume was set to 50 μL, and all measurements were conducted in triplicate. The amount of NH_4_^+^ ions present in the supernatant was determined using an ammonia gas sensing electrode (Monokrystaly). The sulpho—phosphor—vanillin (SPV) colorimetric method was adapted from [26] to quantitatively determine the total intracellular lipid content of the biomass. Measurements were conducted in duplicate, and the reported values represent averages along with the corresponding standard errors.

## 3. Results and Discussion

### 3.1. Construction of Response Surface Models

The experimental runs and their respective process responses are displayed in Table 1. Note that a relatively low (20 g/L) and a very large (150 g/L) initial glucose concentrations, together with their median value (derived according to the central composite DoE), were selected to provide insight into the behavior of cultures under the substrate conditions commonly encountered during fed-batch operations. Conversely, the range of initial ammonium sulfate concentrations in the medium was intentionally limited, ranging from 1 to 5 g/L, with a median value set at 3 g/L. This decision was based on preliminary experiments, which revealed that higher amounts of (NH_4_)_2_SO_4_, up to 10 g/L initially introduced into the culture, led to complete deactivation of the yeast cell population. Throughout the entire set of the experimental trials, the biomass concentration ranged from 3.84 g/L to 10.42 g/L, the lipid content varied from 0.16 g/g to 0.63 g/g, and the residual biomass from 2.7 to 6.6 g/L. The most substantial biomass production was observed in the culture media containing 1 g/L (NH_4_)_2_SO_4_ for all examined glucose levels. Above this threshold, significant growth impairment by about 35% on average (calculated as the averaged reduction in DCW between the two nitrogen levels (3 and 5 g/L) at the three glucose levels) was observed for the three scrutinized glucose levels. The maximum reduction in biomass density (63%) occurred between the experimental points, (85, 1) and (150, 5) g/L, where the abscissa refers to the glucose concentration and the ordinate refers to the (NH_4_)_2_SO_4_ concentration.

Surprisingly, the lipid-free biomass did not consistently increase (i.e., for all the glucose levels) with the enhancement of the initial (NH_4_)_2_SO_4_ concentration in the medium (or a reduction in the C/N ratio) probably indicating culture tolerance to a certain level of the initial glucose loading. This culture behavior against the increased amount of (NH_4_)_2_SO_4_ was more pronounced in relation to the total biomass concentration, where a reverse impact was recorded. However, the diminishing profile of the total biomass concentration, at all three levels of glucose, raising the nitrogen level in the medium, should be inferred together with the interplay between nitrogen and lipid synthesis regulation [27,28].

**Figure 1:**
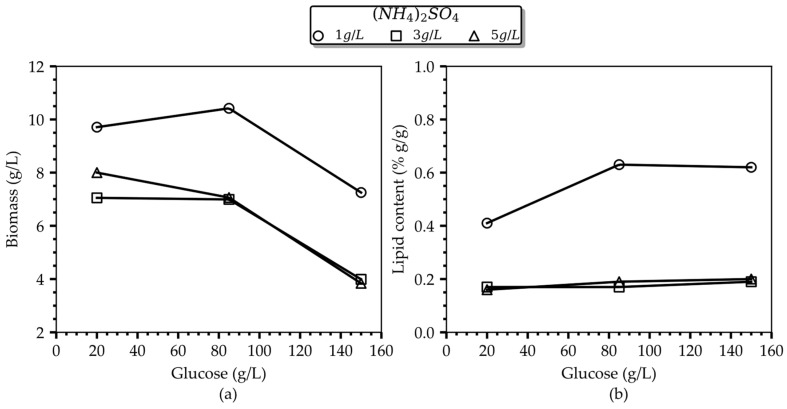
Main effect plots of the initial glucose concentration on the (**a**) total biomass and (**b**) lipid content for the studied (NH_4_)_2_SO_4_ concentrations after 96 h of cultivation.

In Figure 1, the adverse effect of (NH_4_)_2_SO_4_ levels on lipogenesis is readily evident. Specifically, only cells cultured in the presence of 1 g/L (NH_4_)_2_SO_4_ proceeded to the oleogenic phase, while the final oil content per gram of dry biomass increased with increasing glucose concentration until reaching a level of 62–63% *w/w* at 85 g/L glucose. Notably, following a 120 h cultivation period, this noteworthy culture performance was achieved with a relatively modest consumption of both carbon and inorganic nitrogen resources in the medium, as detailed in Table 1 (consumed glucose and (NH_4_)_2_SO_4_ amounted to 24.1 g/L and 0.58 g/L, respectively). Similar lipid levels (i.e., 61% wt.) were accumulated in the culture with the higher glucose concentration (150 g/L), accompanied by roughly equivalent resource consumption (i.e., 22 g/L glucose 0.33 g/L (NH_4_)_2_SO_4_). These findings advocate for an inhibitory effect of glucose beyond a certain threshold, wherein the culture enters an idle phase characterized by significantly reduced metabolic activity and possible different products. Conversely, cells grown with 3 g/L and 5 g/L (NH_4_)_2_SO_4_, independently of the glucose level, reached an average lipid content of 18% wt., suggesting that they did not progress into the oleaginous phase, according to the definition given in [12,13,29]. This recorded outcome in the culture performance seems reasonable for the case of the low initial glucose loading (i.e., 20 g/L), indicating that a glucose depletion in the culture occurred prior to the complete consumption of (NH_4_)_2_SO_4_ in the medium, preventing the transition to the lipogenic phase. However, this notion cannot rationally explain the culture performance in instances of excessive initial glucose loadings (i.e., 85 or 150 g/L). Thus, it is the glucose-inhibition effect that should be blamed, which is developed at glucose levels at around 85 g/L and higher, as evidenced by the detection of both residual glucose and NH_4_^+^ in the fermentation broth, resulting in diminished biomass and lipid yields, as reported in Table 1, and calculated on the basis of the total glucose amount fed to the culture. The authors in [30] reported an inhibitory effect of 60 g/L glucose on the growth of *Cryptococcus* sp. SM5S05. The authors attribute the inhibitory action of glucose on growth to the production of organic compounds of the glycolytic and TCA cycle pathways that result in pH decrease outside the optimal range for growth [30].

The experimental data produced via the central composite design were utilized for the construction of response surface regression models for the three main process variables, i.e., biomass concentration, lipid content, and lipid free biomass concentration. These empirical models are useful tools and can be used for gaining high-level insights into the nonlinear relationships between the dependent and the independent process variables. In this case, three second-order polynomials were fitted to the experimental data and are given (in uncoded units) by Equations (2)–(4) for the biomass concentration, lipid content, and lipid-free biomass concentration, respectively:

(2) $$Biomass=11.67+0.04561X_{1}+3X_{2}-3.56\times 10^{-4}X_{1}^{2}+0.43X_{2}^{2}-3.3\times 10^{-3}X_{1}X_{2},$$

(3) $$Lipid\;Content=0.736+2.62\times 10^{-3}X_{1}+0.3752X_{2}-5\times 10^{-6}X_{1}^{2}+0.052X_{2}^{2}-3.39\times 10^{-4}X_{1}X_{2},$$

(4) $$Lipid\;free\;Biomass=4.097+0.0109X_{1}+1.159X_{2}-1.83\times 10^{-4}X_{1}^{2}+0.1323X_{2}^{2}-0.00120X_{1}X_{2},$$

where, $X_{1}$ and $X_{2}$ refer to the glucose and (NH_4_)_2_SO_4_ levels, respectively. Analysis of variance (ANOVA) was conducted to test the statistical significance of the regression models, and the results are presented in Table 2. The R^2^ values of 0.9884, 0.9671, and 0.9257 for the biomass, lipid content, and lipid-free biomass indicate that the variance of the experimental data is accurately explained by the independent variables in the case of all the response variables. The significance of the three models is further proven by the *p*-values of the model parameters for the three responses (*p* < 0.05). The dependence of the biomass concentration on glucose and nitrogen is described by a full quadratic model as *p* < 0.05 for all the polynomial terms. The linear term of glucose exhibits the maximum contribution to the variance of biomass density, as denoted by its F-value. Amongst the quadratic terms, the inorganic nitrogen concentration contributes the most to the variability of the biomass response. For the lipid content, only the linear terms and the quadratic nitrogen effect were found to exert a statistically significant effect on the response variable (*p* < 0.05). Additionally, only the linear terms and the quadratic glucose term were statistically significant in the case of the lipid-free biomass response.

### 3.2. Optimization of Carbon and Nitrogen Levels in the Culture Medium

**Figure 2:**
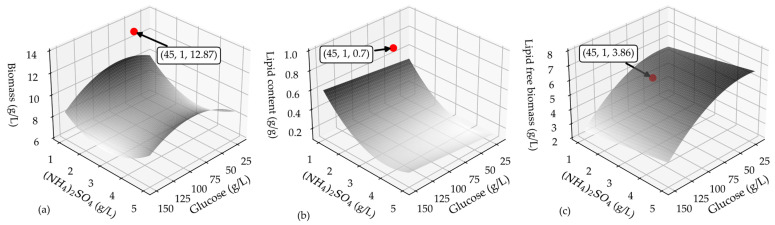
The response surface plot of the (**a**) biomass concentration, (**b**) lipid content, and (**c**) lipid free-biomass concentration as a function of glucose and (NH_4_)_2_SO_4_. The red bullets represent the experimentally measured process performance under the model-derived optimal conditions (text boxes refer to the coordinates of the experimental points, where the *x*-axis represents the glucose concentration and *y*-axis represents the (NH_4_)_2_SO_4_ concentration in the culture medium).

The three regression models, after retaining the significant terms and re-fitting them to the experimental data, were employed to construct the process response surfaces depicted in Figure 2. These graphical representations allow us to investigate the culture conditions, specifically the initial levels of glucose and (NH_4_)_2_SO_4_ that jointly influence the production of the maximum amount of lipids. Considering the discrete steps of culture growth first and lipid synthesis next, which are involved in the non-growth-associated microbial oil production along the cultivation of *T. oleaginosus,* the goal of the maximum lipid amount is pursued through a balanced maximization of the final lipid content and lipid-free (residual) biomass. Such an operational strategy is expected to support future fed-batch fermentation scenarios, where maximizing cell growth in the early stages of fermentation and subsequently transitioning to the oil-accumulation phase (under nitrogen limitation) are commonly implemented. The nonlinear dependence of the biomass, lipid content, and lipid-free biomass on the independent process variables is clearly illustrated in Figure 2. Furthermore, it is apparent that the culture conditions favoring lipid accumulation led to a reduced amount of residual biomass and vice versa, while an extreme point can be identified in the total biomass surface plot.

In pursuit of systematically identifying the optimal medium composition, in terms of carbon and nitrogen sources, to maximize the finally produced amount of lipids, the following objective function (Equation (5)) was formulated. In this equation, the two performance criteria were properly balanced with two weighting factors, $w_{1}=0.4\;\mathrm{and}\;w_{2}=0.6$, to account for their contribution to the overall goal through their concomitant maximization (a head start was given to the ‘lipid free biomass’ considering that in fed-batch cultures intensive lipogenesis can be induced by appropriate feeding policies only on the basis of the lipid-free cell abundance).

(5) $$Obj=w_{1}\cdot Lipid\;content+w_{2}\cdot Lipid\;free\;biomass,$$

(6) $$\begin{array}{c} \underset{X_{1},\;X_{2}\;}{\max}\;\;\;Obj\\ \begin{array}{c} wrt.\;\;\;\;\;\;\;\;\;\;\;\;\;\;\;\;\;\;\;\;\;\;\;\;\;\;\;\;\;\;\;\;regression\;model\;equations:\;\left(3\right),\;\left(4\right)\\ \;\;\;\;\;\;\;\;\;\;\;\;\;\;\;\;\;\;\;\;\;\;\;\;\;\;\;\;\;constraints:\;20\leq X_{1}\leq 150;1\leq X_{2}\leq 5 \end{array} \end{array}$$

The algorithmic solution of the derived optimization problem, mathematically displayed by Equation (6), revealed that the optimal glucose and nitrogen levels were equal to 45 g/L and 1 g/L, respectively. These conditions closely align with the extreme point of the total biomass surface plot.

To assess the validity and reliability of the regression model, the derived optimal conditions were tested experimentally in a batch culture conducted at a flask scale. All culture conditions apart from the initial glucose and ammonium sulfate concentrations were identical to those in the experimental design. Table 3 displays the predicted and the measured values (average of two duplicated runs). Significant deviations in the predictions from the experimental data were observed in terms of all the performance criteria as displayed in Figure 2a–c of the lipid content and consequently lipid-free biomass. Precisely, the measured values of the total biomass concentration and lipid content were larger by approximately 20% and 40%, respectively, than the predicted values, whereas the experimental value of the lipid-free biomass was 27% lower than the respective predicted one. This inconsistency between the regression model and measured values manifests, on the one hand, the deficiency of the derived model developed on the basis of the produced experimental data, and, on the other hand, the inadequately interpreted, so far, role of the C/N ratio. Consequently, four additional experimental runs were cautiously designed and implemented at the flask scale. The chosen values of the factors subjected to scrutiny are displayed in Table 4. Note that the selected initial glucose and ammonium sulfate concentrations ranged from 30 to 60 g/L and 1 to 2 g/L, while their combinations resulted in cases with the same C/N value but substantially different medium compositions (e.g., experimental runs 1 and 16 and 14 and 17).

Remarkably, among the tested culture media, the most substantial improvement in the total lipid amount and the highest glucose-to-lipid yield (0.28 g/g) were recorded when using lower concentrations of the nitrogen source. Through a comparison between the experimental runs 1 and 16, which share the same C/N value (38), it would be reasonable for someone to expect a similar lipid content between the two runs, but enhanced total biomass production for experimental run 16, due to the more generous provision of resources (i.e., 40 g/L glucose and 2 g/L (NH_4_)_2_SO_4_ compared to 20 and 1 g/L, respectively). However, the experimental outcome was entirely different (if not reversed). A similar contrast emerged through a comparison between the experimental runs 14 and 17. Note that in both these sets of experiments, in the cases of the high nitrogen loading (i.e., experiments 16 and 17), a residual amount of 15 g/L of glucose remained at the end. The argument of the glucose-inhibition effect cannot be applied in this case, as for the same glucose concentration of 40 g/L in experiment 15, the total consumption of both resources was recorded, as shown in Table 4. The authors posit that the C/N ratio might be a misleading and misinterpreted process parameter, while the intracellular C- and N-quotas are more indicative of cellular metabolism. These quotas are, in turn, influenced by the absolute concentrations of the respective carbon and nitrogen sources in the medium, rather than their ratio.

**Figure 3:**
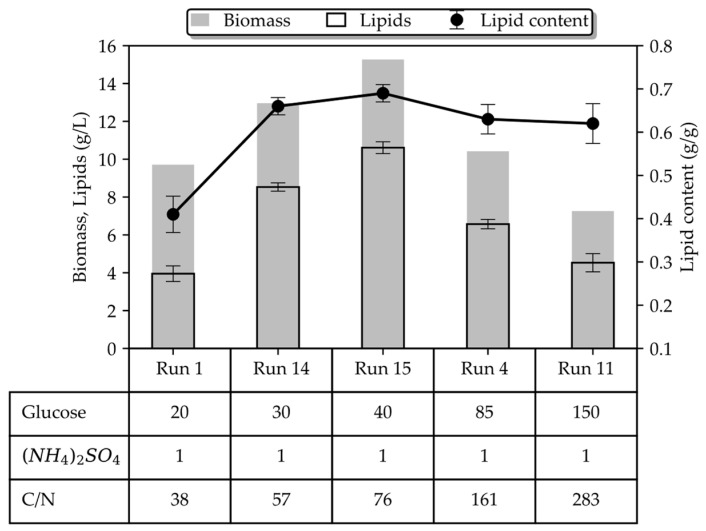
Comparison of the key performance indices between the batch cultures of *T. oleaginosus* grown on glucose and (NH_4_)_2_SO_4_ after 96 h of cultivation. Table entries refer to the initial medium compositions in terms of glucose and (NH_4_)_2_SO_4_ in g/L and the corresponding C/N ratio.

**Figure 4:**
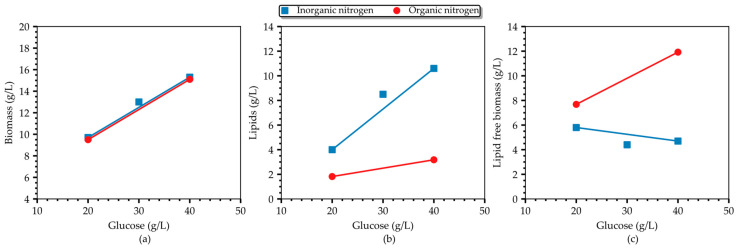
Correlation between the (**a**) biomass concentration, (**b**), lipids concentration, and (**c**) lipid-free biomass concentration and glucose in cultures of *T. oleaginosus* grown on organic (0.26 g/L) and inorganic (0.21 g/L) nitrogen sources. Data from organic nitrogen cultures were obtained from [24].

Figure 3 provides a comparative analysis of batch cultures grown on 1 g/L (NH_4_)_2_SO_4_. Both biomass and lipid concentrations, as well as the lipid content, increase with a rising glucose concentration until the level of 40 g/L, where a maximum is observed (15.27 g/L; 10.6 g/L; 0.69 g/g). However, the inverse correlation between glucose and the lipid-free biomass concentration, for a given level of inorganic nitrogen, was still observed even in the 20–40 g/L range of glucose, typically considered a non-inhibitory region. On the contrary, when almost the same amount of organic nitrogen (in the form of yeast extract) was used in the culture medium, a proportional correlation of the residual biomass with glucose (within the non-inhibitory level) was recorded by the authors in [24] and demonstrated in Figure 4 (the authors used 0.26 g/L organic N, whereas we used 0.21 g/L inorganic N in our study). Interestingly enough, inorganic nitrogen seems to promote lipid accumulation more compared to organic nitrogen (Figure 4b), and as a result, the total biomass is almost equal between cultures grown on inorganic and organic nitrogen. These findings suggest that there may be a combinatory regulatory effect of glucose and NH_4_^+^ on growth with concomitant redirection of the carbon flux to the lipid-synthesis pathway or to another biosynthesis pathway. To the authors’ knowledge, this is the first time this phenotype has been reported in *T. oleaginosus* grown on inorganic nitrogen.

To further enlighten the combinatory regulatory effect of glucose and NH_4_^+^, two batch cultures were conducted in a bioreactor to ensure comprehensive control over culture conditions (e.g., pH, D.O., temperature) and enable efficient monitoring and frequent recording of the fermentation kinetics. The batch cultures were executed employing two distinct media formulations, each characterized by a specific C/N ratio of 38 and 85, respectively. The culture medium compositions in terms of glucose and inorganic nitrogen are given in Table 5, while the remaining culture conditions are detailed in Section 2.2. The selection of the two media (C/N 38 and C/N 85) practically generates the means to duplicate two very distinct flask experiments (where the phenomenon of reduced biomass growth for increasing glucose concentrations was recorded) at the bioreactor scale. The precisely controlled environment of the bioreactor should eliminate the interference of other process variables hampering the reliable explanation and justification of the observed phenomena.

**Figure 5:**
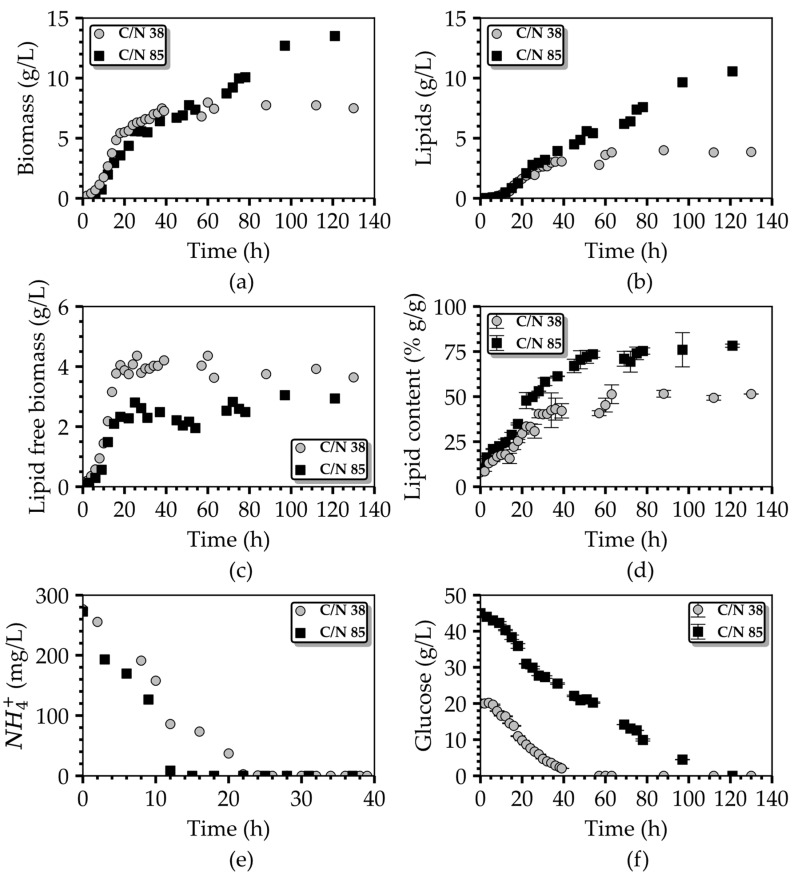
Comparison of the time profiles of the (**a**) biomass concentration, (**b**) lipids concentration, (**c**) lipid free-biomass concentration, (**d**) lipid content, (**e**) NH_4_^+^ concentration, and (**f**) glucose concentration during the batch fermentation of *T. oleaginosus* in a lab-scale bioreactor using two culture media, C/N 38 and C/N 85.

The dynamic progression of these two cultures within the bioreactor is visually represented in Figure 5, showcasing process performance variables and substrate concentrations for both media types. As depicted in Figure 5a, the biomass concentration in the bioreactor utilizing the C/N 38 fermentation medium reached 7.7 g/L at the stationary phase. This value was notably 21% lower than the biomass concentration achieved in the flask culture employing the same medium composition (as detailed in Table 1, first row). A plausible explanation for divergence lies in the variation of the dissolved oxygen level between the two cultivation systems. In the case of the bioreactor scale cultures, DO was constantly maintained at 20% of saturation. In contrast, for the flask-scale experiments, the DO was neither controlled nor measured; however, an aeriation rate of 0.83 vvm was implemented, in contrast to the 0.15 vvm aeriation applied in the bioreactor experiments, to exclude any possibility of O_2_ limitation in the flasks. We postulate that the DO levels in the shake-flask cultures remained considerably above 20% for an extended duration, thereby contributing positively to the extra biomass accumulation [31].

At the end of fermentation in the bioreactor, the lipid concentration and lipid content amounted to 3.86 g/L and 51.4% wt., respectively. This resulted in a glucose-to-lipid yield of 0.19 g/g and maximum lipid productivity of 60 mg L^−1^ h^−1^. Similarly, in the case of the C/N 85 fermentation medium, the values of the biomass, lipid concentration, and lipid content derived for the respective time plots at the end of fermentation were 13.3 g/L, 10.4 g/L, and 78% wt., respectively. These values were notably higher, by 72.7%, 173.7%, and 57%, respectively, compared to those achieved with the C/N 38 culture medium. The performance improvement was also recorded in terms of the lipid yield (0.22 g/g) and lipid volumetric productivity (86 mg L^−1^ h^−1^) in the case of the C/N 85 medium, when contrasted with the C/N 38 one, while in both cases, cultures ended with both C and N sources being entirely consumed by the cells.

Notably, when the C/N 85 culture medium was employed, the consumption of ΝH_4_^+^ was significantly faster than in the case of the C/N 38 medium. Through a comprehensive examination of the kinetic profiles of the ΝH_4_^+^ concentration in the culture medium for both batch cultures (refer to Figure 5e) one can discern that the rate of ΝH_4_^+^ uptake in the C/N 85 culture medium, at 19.5 mg g^−1^ h^−^1, exceeded that of the C/N 38 medium, which stood at 9.3 mg g^−1^ h^−1^. This discrepancy resulted in a swifter depletion of nitrogen in the C/N 85 medium, occurring between the 12 h and 15 h, in contrast to the C/N 38 medium, where nitrogen depletion was completed between the 20 h and 24 h. Nitrogen depletion temporally coincides with the end of the growth phase and the initiation of the lipid-accumulation phase in both cultures, as evidently shown in Figure 5c–e.

These observations explain the prolonged lipid-accumulation phase in the case of the C/N 85 medium, which contributed to the increased oil yields. This phenomenon leads to the hypothesis that an excess of carbon may have induced an upregulation of nitrogen uptake, subsequently leading to the earlier onset of the lipid-accumulation phase. Although both cultures evidently consumed exactly the same amount of nitrogen (Figure 5c), the one conducted with the C/N 85 medium, despite growing in a medium with ample carbon resources in comparison to the C/N 38 medium culture, reached a lower lipid-free biomass concentration than the one employing the C/N 38 medium. Taking into account that the elemental nitrogen quota of the yeast biomass does not vary significantly among different cultivation conditions, we postulate that a portion of nitrogen may have exited the cells in the form of organic molecules, such as amino acids and peptides, and was not assimilated for cellular mass synthesis. This hypothesis is further supported by the analysis aimed at the carbon balance in the two bioreactor-scale experiments, where carbon incorporation in cellular mass and produced CO_2_ through respiration along the entire culture duration were considered. It is revealed that in the culture grown on the C/N 38 medium, only 74% of the carbon can be accounted for. This percentage drops to 60% for the culture with the C/N 85 medium. To be more precise, in these two cultures, there exists an amount of consumed glucose of 5 g/L and 18 g/L, respectively, that cannot be traced (in terms of its carbon content) within the measured culture products. This unaccounted carbon is presumed to have been released from the cells into the broth as extracellular (by)products of yeast metabolism. It is noteworthy that with an increase in the initial glucose concentration, the unaccounted organic carbon also displayed an upward trend. These observations advocate for the existence of side signaling pathway(s) that are activated during the growth phase and lead to diminished biomass production.

In summary, the biomass and lipid production observed in our experimental setups are either comparable to or slightly higher (approximately 2–3 g/L of additional total biomass) than the values reported in the literature for the same strain under analogous culture conditions involving the use of organic nitrogen sources [24,32,33]. Notably, the lipid yield (g_lipid_/g_glucose_) in our study is roughly two-fold greater than that achieved by other researchers. For instance, our setup yielded 0.28 g_lipid_/g_glucose_, as opposed to the 0.12–0.14 g of lipid per gram of glucose achieved by the authors in reference [32] when using NH_4_Cl, asparagine, or L-glutamate as nitrogen sources. A similar trend is apparent when comparing our lipid yield (0.20 g of lipids per gram of glucose) with the yield achieved in reference [24] (0.09 g of lipids per gram of glucose) when yeast extract served as the nitrogen source. It is important to note that these comparisons can only be approximate due to variations in experimental conditions, particularly concerning the quantity and type of nitrogen, as well as the levels of phosphorus [24,32], and other experimental factors, such as the absence of pH control in bioreactor setups [33]. It is worth emphasizing once more that in cultures of *T. oleaginosus*, the lipid yield and consequently the lipid content, considering that the total biomass is roughly similar in all compared cases, appears to be higher when inorganic nitrogen sources are used, rather than organic nitrogen sources. Strikingly, the opposite effect was observed in cultures of *Rhodosporidium toruloides* CBS 14, as explored by the authors in reference [32]. When *R. toruloides* was cultivated with organic nitrogen sources, it accumulated an average of 35% by weight lipids. In contrast, the average lipid accumulation was only 19% by weight when grown with inorganic nitrogen sources [32,34]. This pronounced difference in phenotypes among distinct species of oleaginous yeasts warrants careful consideration, especially in conjunction with the choice of the nitrogen source and other process constraints, as it holds significant implications for the successful scale-up and commercialization of SCOs.

## 4. Conclusions

Concluding this study, the design of experiments and response surface methodology were employed to gain deeper insight into the oleaginous mechanism and the metabolism shifts of the *Trichosporon oleaginosus* yeast, with the prospect of supporting the design of fed-batch cultivation strategies to optimize lipid production in cultures using solely an inorganic nitrogen source. The study achieved maximum lipid production of 10.6 g/L under conditions of 40 g/L glucose and 1 g/L ammonium sulfate, corresponding to an elemental mass C/N ratio of 76. This outcome demonstrated the yeast’s capacity to accumulate intracellular lipids up to 69% *w/w*, with a glucose-to-lipid conversion efficiency of 0.27 g/g. Interestingly, it has been experimentally realized that within a certain range of inorganic nitrogen source concentrations in the culture, an increasing glucose concentration led to a reduction in lipid-free biomass production in favor of enhanced lipid accumulation. This adaptation of the yeast phenotype in varying environmental conditions can be exploited to uncouple growth and lipid production and optimize them sequentially in two-stage cultivation systems.

## Figures and Tables

**Table 1:** Levels of the process variables and experimental results in terms of biomass, lipid, lipid content, lipids, lipid free biomass, biomass, and lipid yield for the optimization of carbon and nitrogen concentrations in the culture medium of *T. oleaginosus*.

	Factors			Performance Criteria							
Exp. Runs	Glucose (g/L)	(NH_4_)_2_SO_4_ (g/L)	C/N	Biomass (g/L)	Lipid Content (% wt.)	Lipid Free Biomass (g/L)	Lipids (g/L)	Y_X/G_ (g/g)	Y_L/G_ (g/g)	Residual G * (g/L)	Residual N * (g/L)
1	20	1	38	9.7	41 ± 4	5.8 ± 0.4	4.0 ± 0.4	0.49	0.20	0.0	0
2	20	3	13	7.1	17 ± 1	5.8 ± 0.1	1.2 ± 0.1	0.35	0.06	0.0	0.42
3	20	5	8	8.0	16 ± 1	6.7 ± 0.1	1.3 ± 0.1	0.40	0.06	0.0	1.89
4	85	1	161	10.4	63 ± 5	3.9 ± 0.5	6.6 ± 0.5	0.12	0.08	60.9	0.42
5	85	3	54	7.06	17 ± 1	5.9 ± 0.6	1.2 ± 0.1	0.08	0.01	57.57	0.76
6	85	3	54	7.30	16 ± 1	6.1 ± 0.6	1.2 ± 0.1	0.09	0.01	59.47	0.88
7	85	3	54	6.65	16 ± 1	5.6 ± 0.5	1.0 ± 0.1	0.08	0.01	62.07	0.99
8	85	3	54	6.96	17 ± 1	5.8 ± 0.6	1.2 ± 0.1	0.08	0.01	63.6	0.98
9	85	3	54	6.99	17 ± 1	5.8 ± 0.6	1.2 ± 0.1	0.08	0.01	61.1	0.90
10	85	5	33	7.1	19 ± 1	5.7 ± 0.1	1.4 ± 0.1	0.08	0.02	60.70	3.21
11	150	1	283	7.3	61 ± 3	2.7 ± 0.2	4.5 ± 0.2	0.05	0.03	138.0	0.67
12	150	3	95	4.0	19 ± 3	3.2 ± 0.0	0.8 ± 0.1	0.03	0.01	136.0	2.02
13	150	5	57	3.8	20 ± 1	3.1 ± 0.1	0.8 ± 0.1	0.03	0.01	134.7	3.95

* G and N refer to glucose and (NH_4_)_2_SO_4_ concentrations, respectively.

**Table 2:** Analysis of variance (ANOVA).

Source		Biomass		Lipid Content		Lipid-Free Biomass	
		R^2^ = 0.9884, Adj. R^2^ = 0.9802		R^2^ = 0.9671, Adj. R^2^ = 0.9436		R^2^ = 0.9257, Adj. R^2^ = 0.8726	
	DF	F-Value	*p*-Value	F-Value	*p*-Value	F-Value	*p*-Value
Model	5	119.64	0.000	41.16	0.000	17.44	0.001
Linear	2	212.70	0.000	63.00	0.000	34.98	0.000
X_1_↝[Glucose]	1	240.69	0.000	7.38	0.030	62.73	0.000
X_2_$↝\left[{\left(NH_{4}\right)}_{2}SO_{4}\right]$	1	184.70	0.000	118.62	0.000	7.23	0.031
Square	2	80.72	0.000	37.64	0.000	8.40	0.014
X_1_^2^	1	96.31	0.000	0.80	0.401	7.23	0.031
X_2_^2^	1	125.44	0.000	69.22	0.000	3.37	0.109
2-Way Interaction	1	11.37	0.012	4.51	0.071	0.42	0.536
X_1_X_2_	1	11.37	0.012	4.51	0.071	0.42	0.536
Error	7	-	-	-	-	-	-
Lack-of-Fit	3	1.39	0.367	91.21	0.000	15.10	0.012
Pure Error	4						
Total	12	Significance level: a = 95%					

**Table 3:** Process performance under the predicted optimal culture condition-comparison of the regression model-based results with the experimental measurements.

Culture Conditions	Process Response	Predicted Value	Measured Value
Glucose, 45 g/L (NH_4_)_2_SO_4_, 1 g/L	Biomass (g/L)	10.68	12.87 ± 0.22
	Lipid content (% g/g)	50	70 ± 1
	Lipid free biomass (g/L)	5.29	3.86 ± 0.04
	Y_X/G_ (g/g)	0.24	0.29
	Y_L/G_ (g/g)	0.12	0.20
	Residual G (g/L)	-	0
	Residual N (g/L)	-	0

**Table 4:** Variable levels and experimental results in terms of the biomass, lipid, lipid content, lipids, lipid-free biomass, biomass, and lipid yield for the complementary set of batch experiments.

	Factors				Performance Criteria						
Exp. Runs	Glucose (g/L)	(NH_4_)_2_SO_4_ (g/L)	C/N	Biomass (g/L)	Lipid Content (% wt.)	Lipid Free Biomass (g/L)	Lipids (g/L)	Y_X/G_ (g/g)	Y_L/G_ (g/g)	Residual G * (g/L)	Residual N * (g/L)
14	30	1	57	13.0	66 ± 1	4.4 ± 0.2	8.5 ± 0.3	0.43	0.28	0.0	0.0
15	40	1	76	15.3	69 ± 2	4.7 ± 0.3	10.6 ± 0.3	0.38	0.27	0.0	0.0
16	40	2	38	8.7	32 ± 4	5.9 ± 0.4	2.8 ± 0.4	0.22	0.07	15	0.0
17	60	2	57	7.7	35 ± 2	5.0 ± 0.1	2.7 ± 0.1	0.13	0.06	15	0.0

* G and N refer to glucose and (NH_4_)_2_SO_4_ concentrations, respectively.

**Table 5:** Composition of the culture medium in terms of the carbon and the nitrogen source for batch cultivations in the 3 L bioreactor.

Run	Glucose (g/L)	(NH_4_)_2_SO_4_ (g/L)	C/N
1	20	1	38
2	45	1	85

## Data Availability

Data are contained within the article.
